# Exploiting survey and online patient experience comments in general practice: validating a fine-tuned language model for automatic sentiment analysis

**DOI:** 10.1093/fampra/cmag071

**Published:** 2026-09-09

**Authors:** Rebecka Maria Norman, Lilja Charlotte Storset, Petter Mæhlum, Hilde Hestad Iversen, Elma Jelin, Erik Velldal, Lilja Øvrelid, Øyvind Bjertnæs

**Affiliations:** Division for Health Services, Norwegian Institute of Public Health, N-0213 Oslo, Norway; Department of Informatics, Section for Machine Learning, Language Technology Group, University of Oslo, N-0316 Oslo, Norway; Department of Informatics, Section for Machine Learning, Language Technology Group, University of Oslo, N-0316 Oslo, Norway; Division for Health Services, Norwegian Institute of Public Health, N-0213 Oslo, Norway; Division for Health Services, Norwegian Institute of Public Health, N-0213 Oslo, Norway; Department of Informatics, Section for Machine Learning, Language Technology Group, University of Oslo, N-0316 Oslo, Norway; Department of Informatics, Section for Machine Learning, Language Technology Group, University of Oslo, N-0316 Oslo, Norway; Division for Health Services, Norwegian Institute of Public Health, N-0213 Oslo, Norway

**Keywords:** primary health care, general practice, patient-centred care, validation study, health care surveys, machine learning

## Abstract

**Background:**

Automated sentiment analysis can be used to analyse free-text patient experience comments, but in health services research, it requires technical performance and methodological validity. Traditional models lack contextual understanding, whereas masked language models (MLMs) enable nuanced sentiment classification. This study is the first to fine-tune an MLM (NorBERT3_large_) on human-annotated data and evaluate the validity of four-category sentiment classifications across survey and online feedback in general practice.

**Objective(s):**

To evaluate a fine-tuned MLM against human-annotated sentiment labels, compare its performance with traditional methods and a large language model, and assess convergent and known-groups validity using survey and online comments.

**Methods:**

NorBERT3_large_ was fine-tuned on human-annotated free-text survey comments. Performance was evaluated using F1-scores. The model classified unseen online data. Construct validity was assessed per COSMIN guidelines through correlations with patient experience scores and subgroup analyses.

**Results:**

The fine-tuned NorBERT3_large_ achieved high F1-scores on held-out survey test data and outperformed traditional models used in prior patient experience research, as well as a zero-shot large language model reference. On unseen online reviews, performance was high in the random sample and strongest for mixed and positive sentiment in the balanced evaluation set, while the rare neutral comments were difficult to classify reliably. Construct validity was supported by expected correlations with patient experience scores and known subgroup differences.

**Conclusion:**

Beyond good classification performance, the findings support automated sentiment analysis as a valid patient experience measure in general practice. The sentiment classifications may complement traditional patient experience measures and help identify areas for quality improvement.

Key messages

- Free-text comments offer insights for evaluating and improving general practice.
- The fine-tuned model accurately classified four sentiment categories.
- Sentiment classifications showed convergent and known-groups validity.
- It outperformed traditional methods and a zero-shot large language model reference.
- Sentiment analysis can function as a complementary patient experience measure.
- The methodology is open and adaptable for use in other countries.

## Introduction

Patient-reported experience measures (PREMs) often include closed- and open-ended questions [1, 2]. While closed-ended survey items can be used for quantitative analysis, open-ended questions allow patients to describe their experiences in their own words [3]. These free-text comments are increasingly valued for research, quality improvement, and clinical feedback [4]. Moreover, the growth of online rating sites has added substantial volumes of detailed patient experience data. However, patient comments frequently mix positive and negative aspects or have a neutral sentiment. In addition, unedited patient feedback data are often varied in grammar and style, including informal phrasing and subjectless sentences [5]. Such unstructured data often remain underused due to the resource demands of manual analysis.

Natural language processing (NLP) enables automated analysis of human language, including sentiment analysis, which identifies subjective opinions, and their polarity and targets [6]. In patient experience research, previous traditional machine learning models such as Naïve Bayes and support vector machines (SVMs) are most commonly used, largely due to their simplicity and compatibility with data governance requirements for sensitive free-text data [7]. However, these models struggle to capture contextual meaning and cannot recognize neutral or mixed sentiment, often forcing nuanced feedback into oversimplified binary categories [3].

In recent years, masked language models (MLMs) [8] based on transformer architectures [9] have improved sentiment classification. MLMs such as BERT are optimized for contextual text understanding, enabling more precise handling of nuanced or implicit sentiment, and allowing for efficient fine-tuning on relatively small datasets [8]. In parallel, instruction-tuned large language models (LLMs) have been proposed as an alternative, as they can be applied in zero- or few-shot settings with minimal or no domain-specific annotation.

Studies have used MLMs and LLMs to classify sentiment in online data, but often without domain-specific human-annotated data or validation beyond classification accuracy [10, 11]. Recent studies on Norwegian patient comments have found that LLM-based zero- and few-shot approaches perform well on simple binary sentiment, but still fall short of human annotators, particularly for neutral and mixed categories [5].

While previous studies have applied BERT-based models to patient feedback [12, 13], these have typically used binary or three-class classification and have not combined multiple data sources. Moreover, most prior studies focus primarily on classification performance and do not assess whether model-classified sentiment constitutes a valid measure of patient experience. Fine-tuning an MLM with annotated data improves the model's ability to interpret nuanced sentiment and better capture patient language [11].

To our knowledge, this is the first study to fine-tune an MLM using a large, domain-specific set of human-annotated data for sentiment classification across both free-text survey responses and online patient feedback, using a four-category scheme: positive, negative, mixed, and neutral. In addition, the study evaluates the construct validity of the sentiment classifications by comparing them with quantitative patient experience scores. This shifts the focus from sentiment classification as a purely technical task to sentiment analysis as a measurement instrument in health services research and quality measurement.

The objectives of this study were to:

1. evaluate the performance of a fine-tuned MLM against human-annotated sentiment labels;
2. compare the MLM's performance with traditional machine learning methods, including NB and SVM, which have been commonly used in previous patient experience research [3], and with a zero-shot instruction-tuned LLM as an additional reference;
3. assess construct validity (convergent and known-groups) using nonannotated survey and online comments linked to patient experience scores.

## Methods

### Survey data

We used data from a national survey of patient experiences with the general practitioner (GP) and the GP practice in Norway conducted in 2021–2022 [14]. The survey included a main sample of 18 861 patients (*n* = 7912 responses) randomly selected from a national sample of GPs. An additional sample of 290 patients was drawn for 50 of the selected GPs (*n* = 5444 responses). The questionnaire comprised five subscales: *Assessment of GP* (8 items), *Coordination* (2 items), *Patient enablement* (3 items), *Accessibility* (2 items), and *Practice* (3 items), as well as an overall item on *General satisfaction with GP*. The last part of the questionnaire included a free-text comment field, where the participant could further elaborate on aspects related to experiences with the GP and the GP's practice [15]. The number of comments was 1632 in the main sample and 1104 in the additional sample.

### Online data

Online reviews were sourced from ‘Legelisten.no’, a Norwegian physician rating website [16]. The web-based questionnaire started with an open-ended question about the overall impression of the GP, followed by a mandatory closed-ended item about the overall assessment of the treatment on a scale from 1 to 5 stars, labelled from ‘very dissatisfied’ to ‘very satisfied’. All published comments and ratings of GPs at Legelisten.no from 26 May 2012, to 15 December 2021, were included in this study (*n* = 76 521).

### Model choice and data governance

The patient comments contain sensitive health-related information and were handled in accordance with strict data protection and governance frameworks. Consequently, raw text data could not be shared with external proprietary services, which ruled out the use of commercial LLMs accessed via external APIs. Hence, we selected an open-source Norwegian BERT-based model (NorBERT3_large_) that could be fine-tuned and evaluated entirely within a secure environment. This ensured compliance with the General Data Protection Regulation (GDPR) requirements while enabling transparent and reproducible model development as well as full control over model versions and updates, which is important when sentiment analysis is intended for repeated use over time.

### The machine learning pipeline

We annotated 2178 comments from two surveys: the GP survey, as described above, and a survey of inpatient experiences with specialist mental health care, conducted in 2020–2022 [17]. Sentiment was labelled positive, negative, mixed, and neutral (no sentiment), at both the comment and sentence levels. Annotation followed a detailed guideline based on NoReC_fine_ (Norwegian Review Corpus fine-grained) [18], adapted to healthcare comments. Annotation was carried out in several rounds, with between two and seven trained annotators participating in each round. Interannotator agreement (IAA) was assessed at regular intervals on held-out sets. Disagreements and problematic cases were discussed in weekly meetings and, where necessary, adjudicated by a senior team member. The guideline was refined iteratively after pilot and test rounds, and earlier labels were updated where revised definitions applied. IAA showed Cohen's *k* values between 0.92 and 0.96, indicating almost perfect agreement [19]. Full details on the annotation process are published in Mæhlum et al [5]. As part of preprocessing, language detection distinguished between Bokmål and Nynorsk. We then fine-tuned NorBERT3_large_ [20], using two human-annotated datasets: (1) the full NorPaC dataset (including inpatient special mental health) and (2) the GP-only dataset. The dataset was split into 80% training, 10% validation, and 10% testing. The distributions of annotated comments across sentiment categories for both datasets are shown in Supplementary Tables A1 and A2. Code and configuration files are available at our GitHub repository [21].

### Model performance

To address Objective 1, model performance was evaluated using F1-scores [22] for each of the four sentiment categories on the held-out test set from NorPaC and the GP dataset. Second, the model classified the new unseen online data. A random sample of 200 comments was annotated by two human annotators, and IAA was assessed using Cohen's *k* [19]. Because of the near-absence of “neutral” examples in the initial evaluation set, we created an additional balanced evaluation sample by annotating a random sample of 50 comments from each category following the same annotation guidelines used to train the model. After resolving disagreements, a consensus was established for each sample and used as a reference for computing F1-scores [22].

### Model comparison

To address Objective 2, we compared NorBERT3_large_ to NB and linear SVM using the same datasets, train–test split, and evaluation metrics. For these comparisons, all performance metrics are based on models trained only on the designated training data and evaluated on held-out test data. In addition, we report zero-shot performance of NorMistral-11B-Thinking, a Norwegian LLM with approximately 11 billion parameters [23], evaluated on the same held-out test sets but not trained on the study data. For the zero-shot evaluation, NorMistral-11B-Thinking was prompted to assign one of the four predefined sentiment labels to each comment. The prompt was designed to clearly specify the task and required output format. The full prompt is provided in Supplementary Table A3. A final model trained on all available annotated data was then applied to the independent online-review dataset (Legelisten.no).

### Sentiment variable

We calculated a sentiment proportion variable at the GP level by determining the percentage of comments in each sentiment category. For each GP, the number of comments in each category was divided by the total number of comments for that GP and multiplied by 100. A linear sentiment variable was also calculated (positive = 1, negative = −1, and mixed = 0), excluding neutral.

### Construct validity testing

To address Objective 3, we assessed convergent and known-groups validity, in line with COSMIN guidelines [24].

Convergent validity is commonly assessed by testing predefined hypotheses about expected correlations with related measures [24]. Previous studies have found significant associations between sentiment classifications from free-text comments and quantitative measures of patient experience [25–28]. The following hypotheses were stated:

*H1: Sentiment differs across GPs in low, medium, and high evaluation groups. More positive sentiment is expected in high-evaluation groups and more negative in low.*

*H2: Positive sentiment is correlated with higher patient experience scores, and negative with lower scores. The strongest correlations are expected with the scales most directly related to the GP, as the free-text field explicitly invites comments on the GP and GP practice.*

First, GPs with fewer than 10 valid comments were excluded [16, 29]. To test H1, we calculated a mean evaluation score for each GP by averaging their subscale scores across the six patient experience scales. GPs were then categorized into three evaluation groups: low, medium, and high, based on tertiles of the mean evaluation score. We compared the distribution of sentiment classifications across these groups by calculating the proportion of comments in each sentiment category. Group differences were tested using the Kruskal–Wallis test. To test H2, Spearman's rho was used to assess the correlation between the sentiment proportion variable and the subscale scores for survey and online data separately.

Known-group validity is assessed by testing expected differences between groups that are known to differ on the construct being measured [24]. Previous studies have found that poorer self-reported physical and mental health, not meeting one's own GP, longer time since last contact with the GP, and shorter duration of time on the GP's list are consistently associated with lower scores on all patient experience scales [14, 30]. The following hypothesis was stated:

*H3: Subgroups with lower patient experience subscale scores will have higher odds of negative sentiment.*

To test H3, we ran multinomial logistic regression models at the patient level. Sentiment category was used as the dependent variable, with positive sentiment as the reference category. Odds ratios, standard errors, and *P* values are reported. *P* < 0.05 was considered significant. A figure with an overview of datasets, analysis, and methods used is available in Supplementary Figure A1.

All models were run in a secure computing environment. Machine learning was conducted in Python (version 3.13). All other analyses were performed using R Studio (R version 4.3.0).

## Results

### Model performance

Table 1 shows the F1-scores for NorBERT3_large_, first trained on the NorPaC dataset (including specialist mental health) and second trained on the GP dataset only. When trained on the GP data, the highest F1-score (average across five runs) was achieved for NorBERT3_large_. Human annotations of 200 online comments (50 per sentiment category) showed high agreement between annotators (Cohen's κ = 0.826, *P* < .001). Table 2 shows the F1-scores for NorBERT3_large_, trained on our annotated GP dataset and evaluated on the online-review dataset (Legelisten.no), using human consensus annotations as the reference standard. In the balanced dataset, the model achieved its highest F1-scores for mixed (87.8%) and positive sentiments (85.2%), and lowest performance for neutral sentiment (43.8%). To illustrate the model's limitations, Supplementary Table A4 presents four representative misclassified comments from the online-review dataset, shown as English paraphrased translations for anonymization.

**Table 1:** F1-scores (%) for sentiment classification models trained on free-text comments from 1142 general practice (2021–2022) and 1036 mental health (2020–2022) survey responses in Norway.

Model	Train/test	Positive	Negative	Neutral	Mixed	Avg. accuracy	Avg. F1
NorBERT3_large_	NorPaC	92.13	86.2	74.48	**80.67**	86.85	83.37 ± 1.46
	GP	**93**.**37**	**90**.**02**	84.29	77.81	**87**.**83**	**86.37** **±** **0.66**
NorMistral-11B-Thinking	NorPaC	82.79	73.50	16.67	18.46	68.95	47.85
	GP	80.37	83.95	**85**.**71**	7.14	73.04	64.30
Naïve Bayes	NorPaC	76.84	61.90	0.00	61.04	66.67	49.95
	GP	85.00	68.29	0.00	55.74	68.70	52.26
Linear SVM	NorPaC	82.69	67.39	31.58	63.87	72.15	61.38
	GP	77.08	66.67	42.86	45.83	65.22	58.11

Results for NorBERT3_large_ represent the average macro F1-score across 5 runs, along with the standard deviations. Results for NorMistral-11B-Thinking are obtained using a zero-shot prompt-based approach. Results for Naïve Bayes and Linear SVM are deterministic. Highest scores in bold.

**Table 2:** F1-scores (%) for GP-trained NorBERT3large model vs. human-annotated consensus in 200 Norwegian online reviews (Legelisten.no, 2012–2021).

Comparison	Positive	Negative	Neutral	Mixed	Average accuracy	Average F1
NorBERT3large vs. human consensus (random sample of 200 comments)	97.30	91.76	^a^	85.71	93.47	91.59
NorBERT3large vs. human consensus (balanced evaluation set, 50 from each class)	85.22	76.42	43.75	87.76	76.5	73.29

F1-scores for the GP-trained NorBERT3large compared to human consensus. The balanced evaluation set comprised 50 comments per sentiment category (200 in total).

^a^The neutral class was excluded due to no examples in the sample and a low frequency in the overall dataset (*n* = 69).

### Model comparison

Table 1 shows F1 scores using NB, linear SVM, and NorMistral-11B-Thinking. All three showed consistently lower performance across sentiment categories compared to NorBERT3_large_, with particularly poor results for the neutral and mixed classes. NorMistral-11B-Thinking performed slightly better than NorBERT3_large_ on the neutral class in the GP domain but did not match its overall performance across categories. For the rest of the analyses (Tables 3–5), we used NorBERT3_large_ fine-tuned on the GP dataset.

**Table 3:** Proportions (%) of sentiment categories across GP evaluation groups, based on GP-level survey data (*n* = 46; 2021–2022), online data (*n* = 2985; 2012–2021), with *P* values for group differences.

Evaluation group	1: low evaluation scale score:	2: medium evaluation scale score	3: high evaluation scale score	Difference
Sentiment	Mean proportion % (SD)	Mean proportion % (SD)	Mean proportion % (SD)	*P* value^a^
Survey data				
Positive	22.1 (11.5)	40.3 (13.2)	61.2 (11.2)	<0.001
Negative	39.4 (13.5)	21.5 (8.2)	11.5 (6.4)	<0.001
Mixed	31.8 (7.7)	29.6 (11.4)	24.1 (9.3)	0.078
Neutral	6.8 (4.5)	8.7 (8.8)	3.2 (3.0)	0.037
*Number of GPs*	*13*	*19*	*14*	
Online data				
Positive	39.6 (12.5)	59.0 (12.1)	76.5 (13.1)	<0.001
Negative	30.9 (12.3)	13.9 (7.3)	3.9 (4.6)	<0.001
Mixed	29.4 (12.8)	26.9 (13.4)	19.6 (12.7)	<0.001
Neutral	0.1 (0.9)	0.1 (0.9)	0.1 (0.7)	0.171
*Number of GPs*	*1010*	*980*	*995*	

Values represent the mean proportion and standard deviation (SD) of comments classified into each sentiment category within each evaluation group.

^a^Group differences were tested using the Kruskal–Wallis test.

**Table 4:** Spearman's rho correlations between patient experience scale scores, online overall GP ratings, and sentiment proportions based on GP-level survey data (*n* = 46; 2021–2022), online data (*n* = 2985; 2012–2021).

Sentiment proportion score	Positive		Negative		Mixed		Neutral	
	*r*	*P* value	r	*P* value	*r*	*P* value	*r*	*P* value
Survey data								
General satisfaction	0.83	<0.001	−0.85	<0.001	−0.26	0.082	−0.37	0.012
Assessment of GP	0.81	<0.001	−0.83	<0.001	−0.28	0.063	−0.34	0.022
Coordination	0.78	<0.001	−0.83	<0.001	−0.34	0.023	−0.17	0.246
Practice	0.72	<0.001	−0.66	<0.001	0.36	0.013	−0.23	0.121
Accessibility	0.72	<0.001	−0.69	<0.001	−0.42	0.004	0.03	0.838
Patient enablement	0.78	<0.001	−0.81	<0.001	−0.33	0.027	−0.29	0.050
Online data								
Overall rating of GP	0.84	<0.001	−0.89	<0.001	−0.32	<0.001	−0.05	0.006

**Table 5:** Odds ratios, standard errors, and *P* values from bivariate multinomial logistic regression of patient characteristics associated with negative, mixed, and neutral sentiment (vs. positive) based on patient-level survey data (n = 2736; Norway, 2021–2022).

Characteristics	N	Negative OR	SE	*P* value	Mixed OR	SE	*P* value	Neutral OR	SE	*P* value
Physical health^a^ (ref: both poor and good)	2730									
Very poor/rather poor		1.09	0.19	0.664	1.13	0.19	0.508	0.52	0.43	0.128
Rather good/very good		**0**.**64**	0.11	<0.001	**0**.**71**	0.11	0.002	0.69	0.19	0.055
Mental health^a^ (ref: both poor and good)	2727									
Very poor/rather poor		**1**.**69**	0.23	0.021	1.28	0.23	0.285	0.67	0.52	0.429
Rather good/very good		**0**.**65**	0.13	<0.001	**0**.**58**	0.12	<0.001	0.77	0.23	0.247
Time since last contact with GP^a^ (ref: <1 month ago)	2728									
1–3 months ago		1.10	0.12	0.422	1.15	0.11	0.210	**1**.**62**	0.22	0.029
4–6 months ago		**1**.**41**	0.14	0.014	0.85	0.15	0.273	**2**.**59**	0.24	<0.001
More than 7 months ago		**1**.**77**	0.17	<0.001	1.37	0.17	0.058	**3**.**20**	0.27	<0.001
Usually meet own GP (ref: yes)	2702									
No		**14**.**05**	0.18	<0.001	**6**.**47**	0.19	<0.001	**6**.**05**	0.27	<0.001
Duration of time on GP's list, years (ref: 0–1)	2736									
2–5		**0**.**53**	0.14	<0.001	**0**.**57**	0.14	<0.001	**0**.**35**	0.24	<0.001
6–15		**0**.**27**	0.14	<0.001	**0**.**41**	0.14	<0.001	**0**.**20**	0.25	<0.001
16+		**0**.**19**	0.16	<0.001	**0**.**29**	0.15	<0.001	**0**.**25**	0.25	<0.001

Results from bivariate multinomial logistic regression models with positive sentiment as the reference category. Odds Ratios (OR), Standard Errors (SE), and *P* values are presented for each sentiment category (Negative, Mixed, Neutral). An OR <1 indicates lower odds of the sentiment category compared to positive sentiment, and OR > 1 indicates higher odds. Statistically significant results are indicated with bold (*P* < 0.05).

^a^Self-reported variables

### Construct validity: convergent validity

After excluding GPs with fewer than 10 comments, the survey dataset comprised 1136 classified comments from 46 GPs. The mean number of comments per GP was 24.7 (SD = 7.9), ranging from 11 to 43 comments. The model classified these comments as *n* = 474 positive, *n* = 264 negative, *n* = 330 mixed, and *n* = 68 neutral.

The online dataset consisted of 60 715 classified comments from 2985 GPs. The mean number of comments per GP was 20.3 (SD = 11.0), ranging from 10 to 186 comments. The model classified these comments as *n* = 33 803 positive, *n* = 10 686 negative, *n* = 16 157 mixed, and *n* = 69 neutral. These datasets, aggregated at the GP level, were used in the following analyses.

Table 3 shows the distribution of sentiment categories across the three evaluation groups. The results largely support H1. Positive sentiment was significantly more frequent in the high-evaluation group and least frequent in the low-evaluation group, with the opposite pattern for negative sentiment. Mixed sentiment was relatively stable across groups in the survey data but significantly more common in the low- and medium-evaluation groups in the online data. Neutral sentiment was generally low in all groups, though it was significantly higher in the low-evaluation group in the survey data; no significant differences were found in the online data.

The results in Table 4 support H2. Positive sentiment was correlated with higher patient experience scale scores, while negative sentiment was negatively correlated with all scale scores. Using survey data, *General Satisfaction* had the strongest positive correlation with positive sentiment and the strongest negative correlation with negative sentiment. Similar patterns were observed for the other scales. Mixed sentiment showed small-to-moderate negative correlations across most scales, with the strongest correlation with *Accessibility*. Neutral sentiment showed weaker and mostly non-significant correlations across the scales. For online data, *Overall Rating of GP* had a strong positive correlation with positive sentiment and a strong negative correlation with negative sentiment. We also conducted correlation analysis using the linear sentiment variable, with similar results. Results are provided in Supplementary Table A5.

### Construct validity: known-groups validity

The patient-level analysis using bivariate multinomial logistic regression models (Table 5) largely supports H3. Not usually meeting one's own GP was the strongest predictor of negative, mixed, and neutral sentiment. A longer time since the last contact with the GP was associated with higher odds of negative and neutral sentiment. Longer duration of time on the GP's list was associated with lower odds of negative, mixed, and neutral sentiment. Better physical and mental health were associated with lower odds of negative and mixed sentiment, while very poor or rather poor mental health significantly increased the odds of negative sentiment. No significant association was found for poor physical health.

## Discussion

This study evaluated the fine-tuned NorBERT3_large_ model for sentiment analysis of patient experience comments, compared its performance with traditional machine learning methods and a contemporary instruction-tuned LLM, and assessed whether model-generated sentiment can be used as a valid measurement instrument through analyses of construct validity.

The fine-tuned NorBERT3_large_ performed excellently on the survey-based data, compared to human-annotated data, and outperformed traditional models commonly used in prior patient experience sentiment analysis studies [7]. As a contemporary reference, we also reported NorMistral's zero-shot performance. In this setting, NorMistral-11B-Thinking achieved reasonable performance on positive and negative sentiment, but overall performance was substantially lower than NorBERT3_large_, particularly in the mixed and neutral categories. This pattern is consistent with previous findings that mixed and neutral sentiment are challenging to distinguish across model types [5, 10, 11]; however, our model achieved high F1-scores across all sentiment categories, including the more challenging mixed and neutral classes, demonstrating that it can reliably identify neutral sentiment when such examples are present in the data. To further assess generalizability, we applied the model to unseen online review data. In the online-review dataset, the neutral class was nearly absent, with only 69 comments predicted as neutral. To enable a fairer evaluation of this class, we created a balanced dataset by annotating 50 comments from each sentiment category. The F1-scores for the balanced dataset were acceptable for all classes except neutral; yet, the average F1-score of NorBERT3_large_ was still considerably higher than in a previous study using Naïve Bayes and two Norwegian large-language models for four-class prediction [5]. Even the neutral class F1-score exceeded the average F1-scores across all classes in that study. The lower F1-score for the neutral class in the online data likely reflects the low number of neutral comments, rather than a model weakness. Online rating platforms are typically dominated by positive or negative opinions, with neutral comments being rare. When a four-class model is applied to data that in practice contains only three classes, performance on the neutral category is expected to be unstable, reflecting the difficulty of classifying a category that is scarcely represented in the data.

Overall, although NorBERT3_large_ did not perform optimally for the neutral category in the online data, the model represents a clear improvement over previously used models in patient experience sentiment analysis [7]. Given the very low frequency and ambiguity of neutral comments, and that including this class lowers the average F1-score without adding much practical value, we recommend using a three-class representation (positive, negative, and mixed) for online rating data.

Compared to previous BERT-based studies in similar health care settings, the model showed improved performance, particularly on negative sentiment. For example, van Buchem et al [12] reported an F1-score of 0.63 for negative sentiment, while our model reached 0.90 on the GP survey data. Osváth et al [13] reported an overall accuracy of 85.9% (binary) and 72.2% (three-class), but did not include a mixed sentiment. Including a mixed category may enhance the model's ability to reflect the complexity of patient feedback.

Beyond classification performance, it is important to assess whether the model's sentiment classifications can be used as meaningful measures of patient experience. We found consistent, theoretically expected correlations between the model's classifications and quantitative patient experience scores, supporting convergent and known-groups validity. First, a clear, statistically significant pattern was observed in the sentiment distribution across GPs in the low, medium, and high evaluation groups. As hypothesized, GPs in the high evaluation group had more positively classified comments, while those in the low group had more negative classifications. Second, a significant correlation was found between sentiment classifications and patient experience scores at the GP level in both survey and online data. Positive sentiment was associated with higher scores, and negative sentiment with lower scores, supporting convergent validity. As hypothesized, the strongest correlations were with the scales assessing aspects directly related to the GP. These findings align with previous research showing associations between free-text sentiment and overall evaluations of care [25]. For example, Dunivin et al [28] found that online reviews correlated with physician trust, communication, and satisfaction.

To our knowledge, this is the first study to examine the known-groups validity of sentiment classifications generated by an MLM in the context of patient experience measurement in general practice. As hypothesized, sentiment varied across patient subgroups in line with previous findings on patient experiences [14, 30]. Patients with shorter duration on the GP's list or those who did not usually see their own GP were more likely to express negative sentiment. These results are in line with previous studies showing that higher continuity of care is associated with better patient outcomes [31–33].

We also found that patients with poorer self-reported mental health had higher odds of negative sentiment, while better mental health was associated with lower odds. This is supported by previous studies showing a positive association between better self-reported health and higher satisfaction with care [33, 34].

To ensure high-quality classifications of patient experience comments, the model required an initial investment in manual annotation. The annotated comments were essential for achieving good performance, validity, transparency, and reproducibility. While instruction-tuned LLMs may reduce annotation effort, their use poses challenges in terms of computational and energy demands. In health services research and quality measurement, sentiment analysis is intended as a measurement instrument to be used over time, stability and suitability for large-scale, repeated use are pivotal considerations. These factors motivated the use of a locally deployable, fine-tuned MLM, which also enables efficient large-scale analysis with modest computational resources.

### Limitations

A limitation is that only about 20% of survey respondents provided free-text comments. While this is similar to other national surveys, those who choose to write comments likely represent a specific subset of patients. Therefore, free-text comments are not generalizable but can offer complementary and in-depth insights that supplement structured survey data. The model was trained and tested on Norwegian-language GP data. This ensured contextual relevance within the Norwegian setting, but its performance in other healthcare services needs to be tested. Further research is required to evaluate its broader applicability. However, the underlying pipeline, including annotation guidelines, fine-tuning approach, and evaluation metrics, is transferable to other countries and datasets, including open online feedback data. This makes the approach potentially relevant for primary care internationally, beyond the Norwegian context.

### Implications and further research

This study is part of a larger project aiming to develop and validate an MLM for polarity- and aspect-based sentiment classification [5]. This study focused on the polarity (positive/negative/neutral/mixed) classification of Norwegian general practice comments, showing that the models perform well at the comment level, with evidence of construct validity at both the individual patient and aggregated GP levels. Few machine learning studies in this context document both technical performance and multiple aspects of validity, despite this being a prerequisite for meaningful use in health services research and quality measurement. Our findings, therefore, extend previous research by demonstrating that strong model performance can be accompanied by evidence of validity across several dimensions.

Previous research has highlighted the value of combining polarity with aspect classification to generate more actionable insights from patient feedback, which can support quality improvement in primary care [11, 35]. Following the promising results of this study, our next step is aspect-based sentiment classification. Building on the polarity results, ongoing annotation of aspects will enable testing the model's ability to differentiate sentiment across aspects such as communication, accessibility, and treatment quality, offering more detailed and valuable information for quality improvement.

Our approach is also consistent with evidence that many clinicians, particularly in primary care, perceive open online feedback as unrepresentative and of limited value for improving services [36, 37]. We have previously shown that there are scientific challenges associated with using quantitative data from Legelisten.no to evaluate quality at the GP level [16], relating to both the quality of the instrument and the generalizability of assessments from small, self-recruited patient samples. The present study showed that the MLM performed technically well on online data but also confirmed the skewed distributions (almost no neutral comments) and the lack of patient-level background variables (e.g. self-reported health, chronic conditions) for assessing relevant aspects of construct validity. As argued previously, it is important for patients, clinicians, and others to interpret online ratings with caution and further develop such sites [16]. The latter also includes adequate item formulations of free-text questions and the inclusion of background variables to assess the performance of machine learning models. The procedures, including guidelines, data annotation, and model fine-tuning, are replicable in other countries using country-specific BERT models, and our work in a low-resource language context demonstrates the feasibility of this approach beyond English and other high-resource languages. We are aiming to fund an international research collaboration across six European countries (Belgium, Czechia, the Netherlands, Norway, Portugal, and Slovenia) on patient-reported outcomes and experiences, including the assessment of generalizability (e.g. health-system specificity), dissemination, and potential application of procedures and models from the current study.

## Conclusions

The fine-tuned NorBERT3_large_ demonstrated strong classification performance, outperforming traditional machine learning methods commonly used in prior patient experience research and showing higher overall performance than a contemporary instruction-tuned LLM used as a zero-shot reference. Sentiment classifications closely aligned with human-annotated data and showed consistent, theoretically expected correlations with quantitative patient experience scores, supporting their use as a valid measurement instrument. As a first step in a larger project, this polarity-based model provides a foundation for further training to support aspect-based analysis and generate more detailed, actionable insights for quality improvement. The sentiment classifications may serve as a complementary measure to traditional PREMs and provide clinically relevant insights to help identify areas for quality improvement in GP practice.

## Supplementary Material

cmag071_Supplementary_Data

## Data Availability

Anonymous survey data are available from the corresponding author on reasonable request. Data from Legelisten.no is restricted to NIPH use; contact Legelisten.no directly for access to their data.
