# Supplementary material for: Exploiting survey and online patient experience comments in general practice: validating a fine-tuned language model for automatic sentiment analysis
Source: Fam Pract. 2026 Sep 9;43(5):cmag071. doi: 10.1093/fampra/cmag071 (PMC13555293; doi:10.1093/fampra/cmag071)
Supplement: Supplementary file 1 [file cmag071_supplementary_data.pdf]

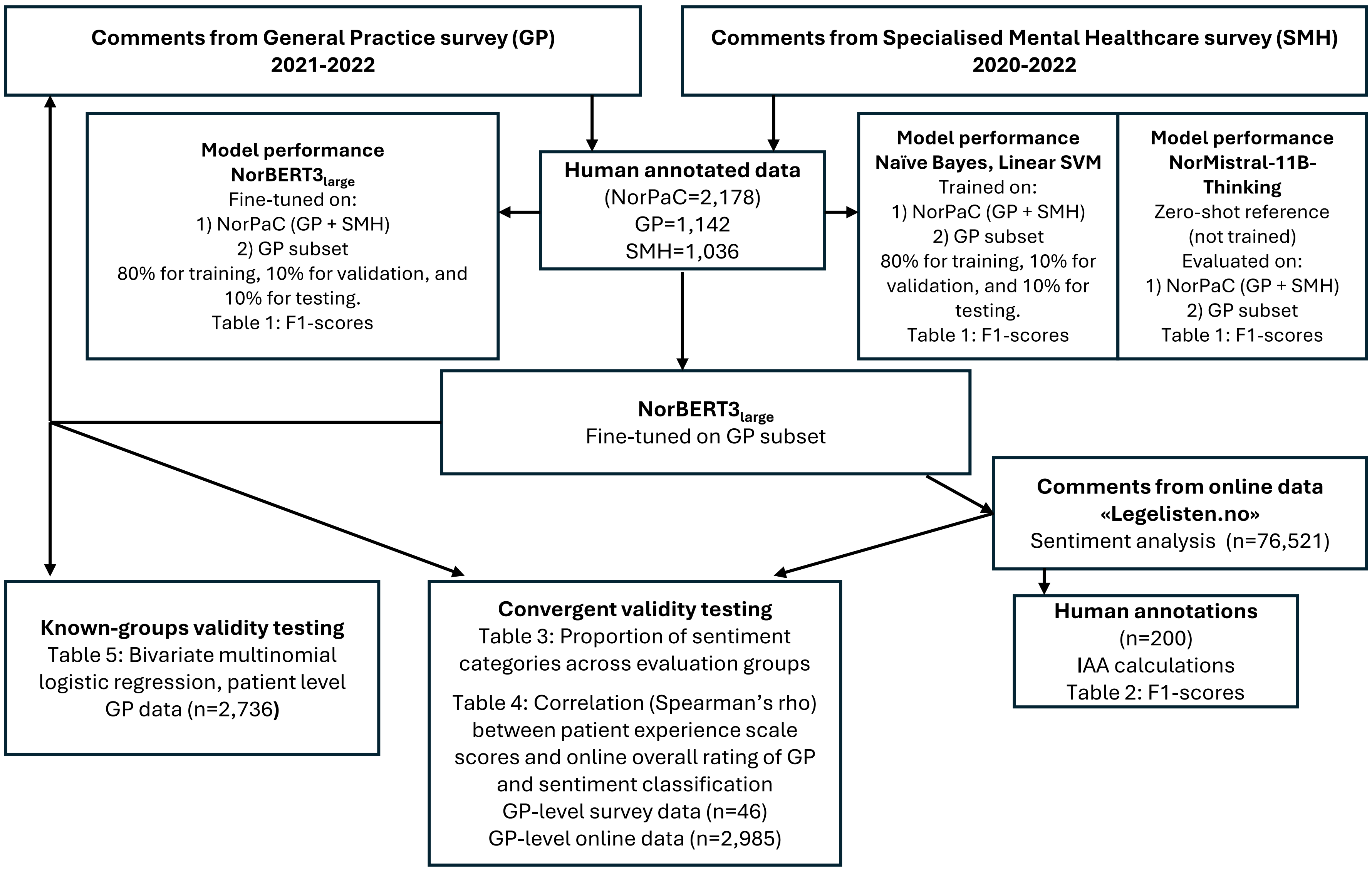

Comments from General Practice survey (GP) 2021-2022
Comments from Specialised Mental Healthcare survey (SMH) 2020-2022
Model performance NorBERT3$_{large}$ Fine-tuned on: 1) NorPaC (GP + SMH) 2) GP subset 80% for training, 10% for validation, and 10% for testing. Table 1: F1-scores
Human annotated data (NorPaC=2,178) GP=1,142 SMH=1,036
Model performance Naïve Bayes, Linear SVM Trained on: 1) NorPaC (GP + SMH) 2) GP subset 80% for training, 10% for validation, and 10% for testing. Table 1: F1-scores
Model performance NorMistral-11B-Thinking Zero-shot reference (not trained) Evaluated on: 1) NorPaC (GP + SMH) 2) GP subset Table 1: F1-scores
NorBERT3$_{large}$ Fine-tuned on GP subset
Comments from online data «Legelisten.no» Sentiment analysis (n=76,521)
Known-groups validity testing Table 5: Bivariate multinomial logistic regression, patient level GP data (n=2,736)
Convergent validity testing Table 3: Proportion of sentiment categories across evaluation groups
Table 4: Correlation (Spearman's rho) between patient experience scale scores and online overall rating of GP and sentiment classification GP-level survey data (n=46) GP-level online data (n=2,985)
Human annotations (n=200) IAA calculations Table 2: F1-scores
